# Analysis of Embedded Optical Interferometry in Transparent Elastic Grating for Optical Detection of Ultrasonic Waves

**DOI:** 10.3390/s21082787

**Published:** 2021-04-15

**Authors:** Chayanisa Sukkasem, Suvicha Sasivimolkul, Phitsini Suvarnaphaet, Suejit Pechprasarn

**Affiliations:** College of Biomedical Engineering, Rangsit University, Pathum Thani 12000, Thailand; chayanisa.s61@rsu.ac.th (C.S.); suvicha.sa61@rsu.ac.th (S.S.); phitsini.s@rsu.ac.th (P.S.)

**Keywords:** ultrasonic detection, optical sensor, sensor enhancement, instrumentation

## Abstract

In this paper, we propose a theoretical framework to explain how the transparent elastic grating structure can be employed to enhance the mechanical and optical properties for ultrasonic detection. Incident ultrasonic waves can compress the flexible material, where the change in thickness of the elastic film can be measured through an optical interferometer. Herein, the polydimethylsiloxane (PDMS) was employed in the design of a thin film grating pattern. The PDMS grating with the grating period shorter than the ultrasound wavelength allowed the ultrasound to be coupled into surface acoustic wave (SAW) mode. The grating gaps provided spaces for the PDMS grating to be compressed when the ultrasound illuminated on it. This grating pattern can provide an embedded thin film based optical interferometer through Fabry–Perot resonant modes. Several optical thin film-based technologies for ultrasonic detection were compared. The proposed elastic grating gave rise to higher sensitivity to ultrasonic detection than a surface plasmon resonance-based sensor, a uniform PDMS thin film, a PDMS sensor with shearing interference, and a conventional Fabry–Perot-based sensor. The PDMS grating achieved the enhancement of sensitivity up to 1.3 × 10^−5^ Pa^−1^ and figure of merit of 1.4 × 10^−5^ Pa^−1^ which were higher than those of conventional Fabry–Perot structure by 7 times and 4 times, respectively.

## 1. Introduction

Ultrasound is a technology commonly used in various applications, such as in medical diagnosis, medical treatments [1] and photo-acoustic imaging (PI) [2]. Typically, a piezoelectric transducer is employed in ultrasonic detection. However, the device has several limitations, including sensitivity, detection bandwidth [3], long working distance, bulky size, and lacking scalability [4].

**Figure 1:**
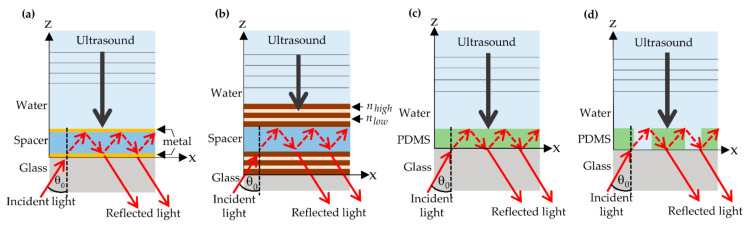
An elastic spacer is (**a**) sandwiched by bi-layered metallic films, (**b**) coated by Bragg reflectors, (**c**) using polydimethylsiloxane (PDMS) thin film and (**d**) using PDMS grating.

High-sensitivity optical sensors are also investigated for their applications in ultrasonic detection, such as micro-ring resonators [4,5,6,7,8], fiber-based sensors [9,10,11,12], thin film sensors [13,14,15,16]. In the micro-ring resonators and the fiber-based sensors, the acoustic wave is detected by measuring the change of the refractiverefractive index (*n*) and the change in the physical structure introduced by an ultrasonic illumination. These techniques provide a phase shift in the resonant condition and transmittance of optical fiber. Generally, the ring resonator is limited due to the ultrasonic bandwidth depending on the ring’s size, and it requires a sophisticated fabrication process. There are two types of widely employed Fabry–Perot (FP) structures. Firstly, an elastic spacer is sandwiched by two thin metallic films [15] to provide a lossy optical system. Secondly, the spacer is coated by two Bragg reflectors [14,16,17]. The FP resonant modes detect the ultrasonic waves by measuring the change in the optical phase shift of the elastic spacer [13], which is squeezed by the incident ultrasound, as shown in Figure 1a,b.

Learkthanakhachon et al. [18] recently introduced the polydimethylsiloxane (PDMS) thin film sensor detecting ultrasonic waves through a shearing interferometer shown in Figure 1c. The uniqueness of PDMS film is able to provide high sensitivity, small size, and transparency, allowing direct integration to an optical imaging system. This opens up a new avenue for multi-modal imaging and simultaneous imaging of optical and mechanical properties.

Here, we propose a theoretical study of a transparent PDMS thin film grating structure and provide a framework to explain how a PDMS grating, as shown in Figure 1d, responds to optical and ultrasonic illuminations. The grating can provide ultrasensitive FP modes and more extensive compression leading to an enhancement in ultrasonic detection in term of sensitivity and figure of merit (*FOM*). To the best of the authors’ knowledge, this elastic grating structure for ultrasonic detection has never been studied and reported before in the literature.

## 2. Materials and Methods

### 2.1. Material Compression under Ultrasonic Illumination

**Figure 2:**
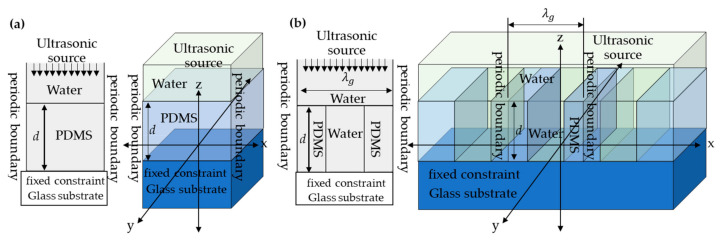
Shows schematic diagrams of (**a**) PDMS thin film model and (**b**) PDMS grating model.

The finite element method (FEM) in COMSOL Multiphysics 5.3a was employed to compute the mechanical responses of schematic models shown in Figure 2. Figure 2a shows the schematic diagram for the uniform PDMS film with the film thickness, *d*, while Figure 2b showed the PDMS grating model with the height of grating (the film thickness), *d*, the grating period, λ*_g_*, and the grating fill factor, *FF*. The COMSOL simulation was based on the acoustic-solid interaction model. The ultrasonic wave radiating at 2 MHz traveled through a water medium and illuminated onto the PDMS structure. The Helmholtz equation [19] explained the acoustic pressure and the mechanical deformations, and the mechanical properties can be calculated by solving Navier’s equations [19]. For the FEM boundary conditions, the left and right boundaries were the periodic boundary condition. The top of the model was the incident ultrasonic plane wave radiation. The bottom boundary condition was a fixed constraint. All reported FEM results in this manuscript were computed with the mesh size of 15–100 nm to ensure that convergence was achieved.

At 2 MHz ultrasonic frequency, Young’s modulus, *E*, of the PDMS was reported 123.4 MPa [18], and Poisson’s ratio was 0.43 [20]. The mechanical properties, such as Young’s modulus of PDMS, depend on the ultrasonic wave’s central frequency [21]. The frequency is a standard frequency for ultrasonic medical imaging and usually investigated in the literature, allowing results obtained in this study to be compared with other ultrasonic detection techniques in the literature.

### 2.2. Optical Reflectance Calculation

The optical reflectance response of the proposed PDMS grating was calculated using rigorous coupled-wave analysisrcwa (RCWA) [22], and the uniform PDMS layer was calculated using Fresnel equations and the transfer matrix approach [23], which are in-house developed under MATLAB R2018a utilizing parallel computing.

**Figure 3:**
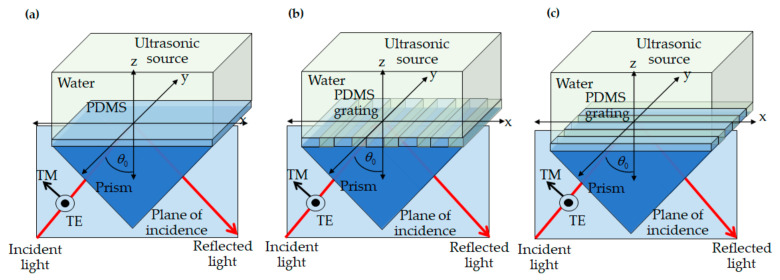
Shows incident transverse magnetic (TM) and transverse electric (TE) polarized light on (**a**) uniform PDMS thin film, (**b**) PDMS grating in case of incident plane perpendicular to the grating stripes and (**c**) PDMS grating in case of incident plane parallel to the grating stripes.

The optical simulation parameters conceptualize a linearly polarized incident light at 685 nm wavelength, λ_0_, illuminating with an oblique incident angle, *θ*_0_, from the bottom as depicted in Figure 3. The reason for choosing the wavelength was to make a fair comparison between the uniform PDMS film and the surface plasmon resonance sensing performance reported in Learkthanakhachon et al. [18].

Two incident polarization states were investigated in this study: the TE and TM. For the uniform PDMS films, the two polarization states are defined as shown in Figure 3a; meanwhile, the grating orientation was also investigated. The two polarizations relative to the grating directions were defined as shown in Figure 3b,c. Figure 3b showed the plane of incidence is perpendicular to the grating stripes, whereas Figure 3c showed the plane of incidence is parallel to the grating stripes. The structure comprised a glass prism with the refractive index, *n*_0_, of 1.52, *n_pdms_* with its refractive index of 1.43 [24], and *n_water_* of 1.33.

### 2.3. Non-Linear Response of the FP Modes and Mathematical Transform for Linearization

A linear response is a preferred sensorgram response [25]. Several methods have been introduced in the literature to transform a non-linear sensorgram to a linear response, including using a mathematical model [26], an additional sensor reader for converting output signal [27], and a neural network [28] to work out the underlining relationship between measurand and sensor output. For the FP mode, they exhibited a non-linear response to the ultrasound. A degree 3 polynomial curve fitting Equation (1) was employed as the sensorgram’s transformation function. Note that the equation does not change the signal-to-noise ratio as it maintains the intensity of the transformed signal to the same level as the signal before the transformation, and it will be shown later that the optical responses from the sensor follow the model with a high coefficient of determination (*R*^2^).

(1) $$R_{T}=aR^{3}+bR^{2}+cR+d$$

where *R_T_* is the reflectance after transformation using Equation (1). *a*, *b*, *c* and *d* are the coefficients in the polynomial equation. *R* is the reflectance calculated using Fresnel equations and the RCWA.

### 2.4. Comparative Sensor’s Performance Parameters

The performance parameters for comparing the structures include sensitivity, *FOM*, detectable range, and *FOM*× detectable range product.

The sensitivity is defined as the sensor responsivity in transformed optical reflectance due to the ultrasonic pressure, which is the ratio between the difference in transformed reflectance (*dR_T_*) over the change in acoustic pressure (*dP*) as shown in Equation (2), assuming that the sensor has a linear response.

(2) $$Sensitivity=\left|\frac{dR_{T}}{dP}\right|=\left|\frac{R_{T,UL}-R_{T,0}}{P}\right|$$

where *R_T,UL_* refers to the transformed reflectance under an ultrasonic pressure loading and *R_T,_*_0_ is the transformed reflectance without an acoustic wave loading.

The *FOM* is defined as the ratio between the sensitivity and the *R_T_*_,0_ as expressed in Equation (3).

(3) $$FOM=\left|\frac{Sensitivity}{R_{T,0}}\right|$$

The detectable pressure range (*α*) is defined as the acoustic pressure range that the sensor can provide a linear responsivity.

The *FOM*× detectable range product (*FOM* × *α*) is to study and compare the relationship between the *FOM* and the detection range for ultrasonic sensing platforms. It is well-established that there is a trade-off between sensitivity and the detection limit [29,30]. The *FOM* is the parameter that has included the sensitivity, and it takes into account the optical power of the signal into consideration.

## 3. Results and Discussion

### 3.1. Ultrasonic Detection Using the Uniform PDMS Thin Film

**Figure 4:**
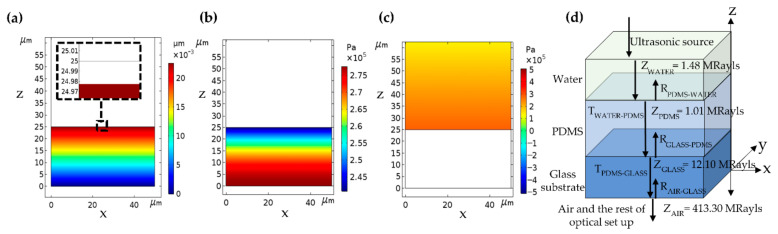
Shows (**a**) the compression, (**b**) the pressure distribution inside the PDMS, (**c**) the pressure profile of incident ultrasonic wave with frequency of 2 MHz and amplitude of 500 kPa, and (**d**) acoustic impedances, reflectance, and transmittance of the uniform PDMS structure.

The FEM calculation shows that the ultrasonic wave with the amplitude of 500 kPa can uniformly compress a 25-μm-thick PDMS thin film by 22 nm in root mean square (RMS) compression, as shown in Figure 4a. Note that for the typical medical ultrasonic imaging, the ultrasonic pressure is around 14 kPa to 650 kPa [31]. The relationship between the thickness compression and the acoustic pressure is linear, and the RMS compression of 4.4 × 10^−5^ nm/Pa, which agrees with the compression experimentally measured and reported in the literature [18].

#### 3.1.1. Mechanical Responses of the Uniform PDMS Layer to the Incident Ultrasonic Wave

It has been established that the change in the refractive index of the compressible material is around 10^−10^ RIU/Pa [6], which is relatively small and neglected in this study. Note that RIU is the refractive index unit. The range of the pressure inside the PDMS was from 2.4 × 10^5^ kPa to 2.8 × 10^5^ kPa, as shown in Figure 4b, and equivalent to 3.7 × 10^−7^ RIU. Optical simulation using the pressure contour, as shown in Figure 4b, and their corresponding refractive indices for the PDMS were compared to when the refractive index of PDMS was treated as a bulk material with the uniform refractive index of 1.43. The two cases’ optical responses were identical and not shown here to save space. The ultrasonic pressure in the water medium corresponded to the illuminating ultrasonic pressure, as shown in Figure 4c.

The distinct separation between the ultrasonic and optical illuminations allows the optical measurement to be carried with little vibration due to the ultrasonic source because of the glass substrate’s acoustic impedances, the elastic layer, the coupling water, and the ambient are very different. The acoustic impedances of the glass substrate, the PDMS at 2 MHz incident ultrasound, the water at 20 °C and the air at 20 °C are 12.10 MRayls, 1.01 MRayls [32], 1.48 MRayls [33] and 413.30 Rayls [34], respectively, as shown in Figure 4d. The glass to the air interface transmittance, the PDMS to the glass interface transmittance, and the water to PDMS interface transmittance were 1 × 10^−4^, 2.7 × 10^−1^ and 9.5 × 10^−1^, respectively. Therefore, the optical system does not suffer from microphonic vibration due to the ultrasonic source. The 95% of sound energy can efficiently compress the PDMS at the water to the PDMS interface thanks to their similar acoustic impedances. However, 26% of ultrasonic energy penetrate the PDMS layer, and, of course, this can cause microphonic vibration of the sensor substrate. A well-known optics practice can overcome the microphonic issue, a common path interferometer [35]. The interferometer’s two optical beams propagate along the same optical path and suffer the same amount of microphonic vibration leading to the same phase shift and suppressing the microphonic effect.

#### 3.1.2. Embedded Fabry–Perot Interferometer Formed by the Two Interfaces of PDMS Layer

**Figure 5:**
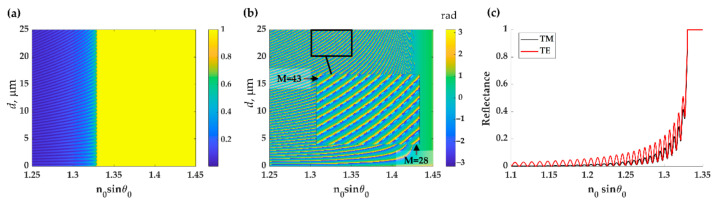
Shows (**a**) contour of reflectance and (**b**) phase responses of PDMS thin film by varying thicknesses *d* for TE polarization, and (**c**) reflectance responses of 25-μm-thick PDMS thin film for TE (red curve) and TM (black curve) polarizations.

Figure 5 shows the uniform PDMS layer’s optical responses when the PDMS thickness *d* is varied from 0 μm to 25 μm with TE polarized light. The FP reflectance dips’ intensity excited by the TE polarization had higher intensity contrast than the TM polarization dips due to the reflection’s nature, polarization-dependent [36], as shown in Figure 5c. Therefore, the TM polarization results for later cases are omitted to save space. Figure 5a,c show that FP modes for both polarizations appear below the critical angle, *n*_0_*sinθ*_0_ of 1.33. The PDMS thin film formed an FP resonant cavity [13] and the two interfaces of the PDMS provide reflected beams as depicted in Figure 1c. No FP reflectance dips appear in the reflectance between the two critical angles of the water and PDMS, *n*_0_*sinθ*_0_ of 1.33 and 1.43, as shown in Figure 5a. However, the FP mode signature is present in the phase response, as shown in Figure 5b. Phase measurement techniques, such as an interferometer, can be employed for sensing applications with ultrasensitivity [37]. However, this, of course, requires sophisticated optical interferometer instrumentation [38] and a vibration isolation system [39]. The inset of the Figure 5b shows a close-up response of the thickness *d* between 20 μm to 25 μm, and the dashed light blue curves highlight the FP mode positions calculated using asymmetric FP cavity mode condition [40] in Equation (4) from the mode number *M* of 28 to 43. The resonant dips in Figure 5 are the FP modes formed by the PDMS layer, and these agree with the FP mode positions calculated using Equation (4).

(4) $$2k_{z,cavity}d+\phi_{upper}+\phi_{lower}=2\pi M$$

where $k_{z,cavity}$ is the wave vector along z-direction in the PDMS material, which is given by $\sqrt{{\left(\frac{2\pi n_{PDMS}}{\lambda_{0}}\right)}^{2}-{\left(\frac{2\pi n_{0}sin\;\theta_{0}}{\lambda_{0}}\right)}^{2}}$. $\phi_{upper}\;$ and $\phi_{lower}$ are the phases of reflection coefficients calculated using Fresnel equations between PDMS to water interface and PDMS to glass interface, respectively [41,42].

#### 3.1.3. Sensorgram of the Uniform PDMS for Ultrasonic Detection

**Figure 6:**
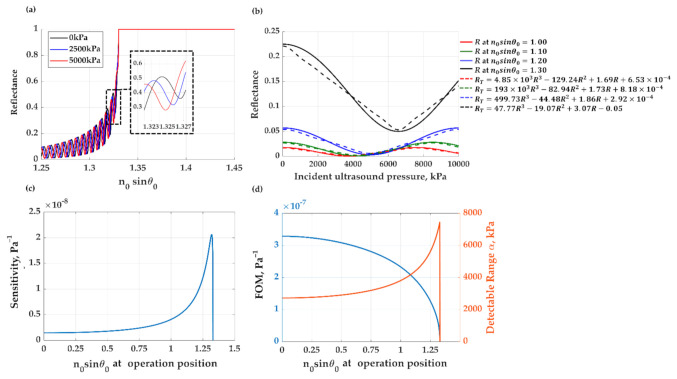
Shows (**a**) reflectance responses of 25-μm-thick PDMS thin film under ultrasound loading with 0 (black curve), 2500 (blue curve) and 5000 kPa (red curve), (**b**) reflectance responses, *R*, and their linearization transformation, *R_T_*, at four operation position, *n*_0_*sinθ*_0_, with R^2^ of 1.0, 1.0, 1.0, and 1.0, respectively. The performance of (**c**) sensitivity and (**d**) the figure of merit (*FOM*) and the detectable pressure range *α* for ultrasonic detection of PDMS thin film for different operation positions.

The PDMS compression due to the incident ultrasound can be measured through the change in FP resonant conditions, as shown in Figure 6a. Figure 6b shows the uniform PDMS layer’s sensorgrams at four operating incident angles of *n*_0_*sinθ*_0_ of 1.00, 1.10, 1.20, and 1.30 responding to ultrasonic pressure levels. The solid curves show the raw reflectance response from the PDMS layer, and the dashed curves show the transformed reflectance using the Equation (1). The transformation is to linearize the sensorgram response, as described in Section 2.3. Figure 6c shows the sensitivity of each operating positions *n*_0_*sinθ*_0_. The maximum sensitivity of 2.1 × 10^−8^ Pa^−1^ and the broadest detectable range of 7130 kPa occurred at the *n*_0_*sinθ*_0_ of 1.32. The maximum *FOM* of 3.3 × 10^−7^ Pa^−1^ was at the operating position, *n*_0_*sinθ*_0_, of 0 (normal incidence) and decreased as the incident angle increased. FP structures usually employ normal incidence due to the high *FOM* [13]; however, at the expense of the detection range and sensitivity [43]. This leads to the classic trade-off between sensitivity and detection range. The *FOM × α* over all the incident angles were around one and allowed the FP modes in the PDMS thin film.

Note that for the uniform PDMS film, the reflectance was below 0.5, leading to a lower amount of light and a more inadequate signal-to-noise ratio than modes appearing beyond the critical angle through the total internal reflection discussed in the later section.

### 3.2. Ultrasound Detection Using PDMS Grating

#### 3.2.1. Mechanical Responses of the PDMS Grating to the Incident Ultrasonic Wave

**Figure 7:**
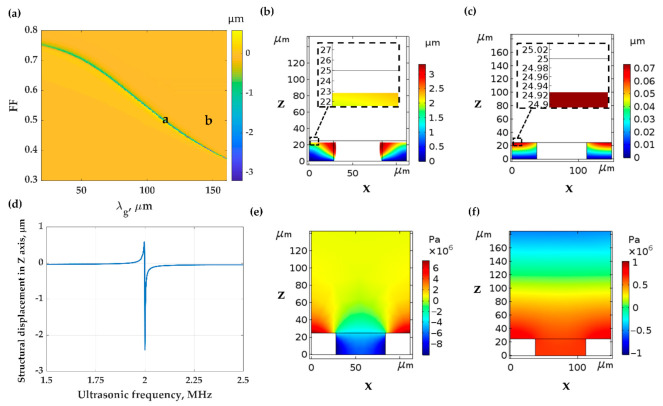
(**a**) Contour of compression with varying grating feature, *λ_g_* and *FF*. The label ‘a’ is the PDMS grating with *λ_g_* of 112.2 μm, *FF* of 0.5 and *d* of 25 μm, and the label ‘b’ is the PDMS grating with *λ_g_* of 150.0 μm, *FF* of 0.5 and *d* of 25 μm. Compression of the PDMS grating (**b**) for ‘a’ and (**c**) for ‘b’ under the 2 MHz ultrasonic wave with the amplitude of 500 kPa. (**d**) Compression of the PDMS grating for ‘a’ under the varying ultrasonic frequency. Pressure distribution of the medium when the PDMS grating with feature of (**e**) ‘a’ and (**f**) ‘b’ was compressed by the 2 MHz ultrasonic wave with the amplitude of 500 kPa.

The PDMS grating is employed as a light diffractor providing a phase-matching condition for the FP modes between the two critical angles of the water and PDMS *n*_0_*sinθ*_0_ of 1.33 and 1.43. These FP modes can be observed with no need for an interferometer. The PDMS grating also provides additional compressibility compared to the uniform PDMS because of the gaps between the grating grooves and the guided surface acoustic wave (SAW) mode, as shown in Figure 7a. Figure 7b shows the compression by FEM simulation of PDMS grating with *λ_g_* of 112.2 μm, *FF* of 0.5 and *d* of 25 μm labeled as the position ‘a’ in Figure 7a. The grating can provide additional compression, and the illuminated ultrasonic wave can also be coupled to a SAW mode [44,45], forming a standing wave pattern on the grating surface, as shown in Figure 7e. The SAW mode enhanced the pressure exerted on the grating surface, improving the thickness compression to 4.8 × 10^−3^ nm/Pa. It has been very well-established that the SAW mode’s coupling condition depends on the frequency of the incident ultrasonic as shown in Figure 7d and grating features including *λ_g_* and *FF* as depicted in Figure 7a [46]. Figure 7c shows the compression by FEM simulation of PDMS grating with *λ_g_* of 150 μm, *FF* of 0.5 and *d* of 25 μm labeled as the position ‘b’ in Figure 7a. The grating can provide the thickness compression of 1.4 × 10^−4^ nm/Pa without SAW mode. The compression is higher than the uniform PDMS layer due to the grooves’ gap space.

#### 3.2.2. Fabry–Perot Modes Formed by Reflections from Each of the Two Interfaces and Diffraction Provided by the PDMS Grating

**Figure 8:**
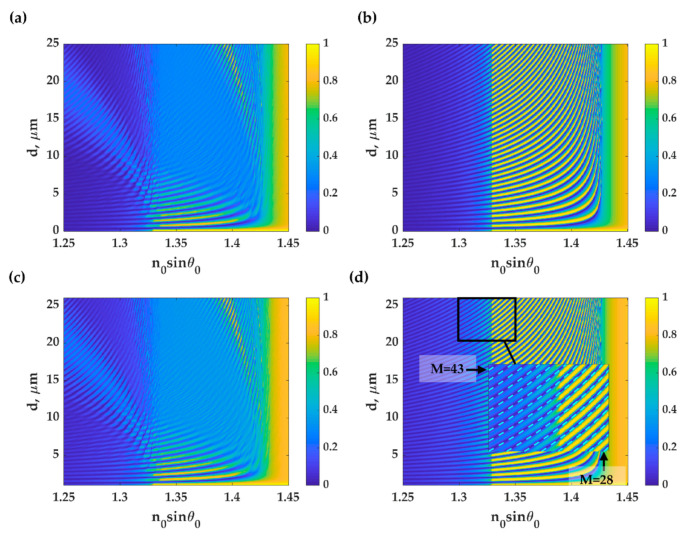
Reflectance responses for the PDMS grating with varying thickness, *d*, *λ_g_* of 112.2 and FF of 0.5. TM polarization optical illumination on the PDMS grating with their (**a**) incident plane perpendicular to the grating stripes and (**b**) incident plane parallel to the grating stripes. TE polarization optical illumination on the PDMS grating with their (**c**) incident plane perpendicular to the grating stripes and (**d**) incident plane parallel to the grating stripes.

Figure 8 shows the reflectance from the PDMS grating with varying thickness *d*, *λ_g_* of 112.2, and *FF* of 0.5 for both TM and TE polarizations and the two grating orientations described in Section 2.2. Figure 8a,c show FP responses and eigenmodes inside the grating due to the phase-matching condition of the grating vectors along the incident plane, whereas, for the other two cases shown in Figure 8b,d, the reflectance only contains the FP modes; the eigenmodes inside the grating were perpendicular to the plane of incidence. The inset in Figure 8d shows the FP mode positions calculated using the asymmetric FP cavity mode condition described in Equation (4) for the M order of 28 to 43. The modes observed in the reflectance contours between *n*_0_*sinθ*_0_ of 1.33 to 1.43 are the FP modes, which cannot be seen in the uniform PDMS case, in Figure 5a.

**Figure 9:**
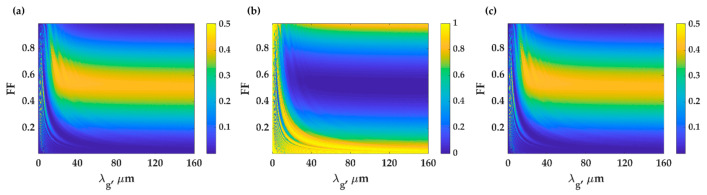
Reflectance responses for PDMS grating with varying *λ_g_* and FF for TE polarization at *n*_0_*sinθ*_0_ of 1.33 for diffraction efficiencies at (**a**) −1st order, (**b**) 0th order and (**c**) 1st order.

Figure 9 shows diffraction efficiencies of the −1st, the 0th and 1st order diffractions of the PDMS grating when illuminated with the optical incident angle, *n*_0_*sinθ*_0_, of 1.33, where the minimum reflectance of 0. For the uniform PDMS layer, *FF* of 1.00, the dif-fraction efficiency of the 0th is 1, as shown in Figure 9b, and the other orders are 0 as shown in Figure 9a,c. The FP modes cannot be seen in the reflectance. However, for the large grating period (non-subwavelength region), the reflectance of the 0th order is 0, as shown in Figure 9b, whereas the energy is distributed to the other two diffracted orders as shown in Figure 9a,c. 

**Figure 10:**
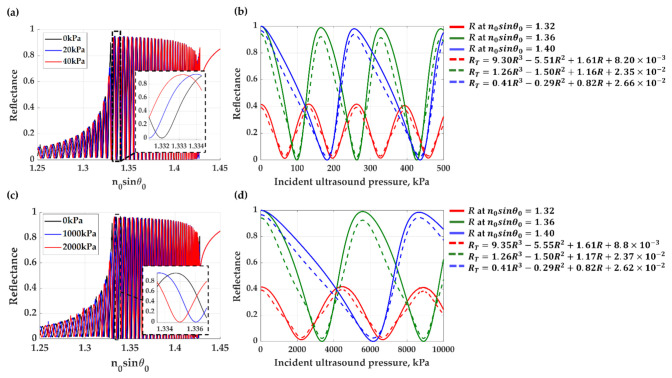
(**a**) Reflectance responses of PDMS grating in SAW mode under ultrasound loading of 0 (black curve), 20 kPa (blue curve), and 40 kPa (red curve) and (**b**) reflectance responses, *R*, and their linearization transformation, *R_T_*, at three operation position, *n_0_sinθ*_0_, with *R*^2^ of 1.0, 1.0 and 1.0, respectively. (**c**) Reflectance responses of PDMS grating without SAW mode under ultrasound loading of 0 (black curve), 1000 kPa (blue curve) and 2000 kPa (red curve) and (**d**) reflectance responses, *R*, and their linearization transformation, *R_T_*, at three operation position, *n_0_sinθ*_0_, with *R*^2^ of 1.0, 1.0 and 1.0, respectively.

Figure 10a,c show the two PDMS gratings’ reflectance responses with *λ_g_* of 112.2 μm and *FF* of 0.5 (in the SAW mode coupling) and with *λ_g_* of 150 μm and *FF* of 0.5 (without the SAW mode coupling) when compressed by the incident ultrasound. Figure 10b,d show sensorgrams of the two gratings for three operating positions *n*_0_*sinθ*_0_ of 1.32 (red curves), 1.36 (green curves) and 1.40 (blue curves); the solid curves represent the reflectance calculated using RCWA, and the dashed lines are the reflectance after linearization using the Equation (1).

#### 3.2.3. Sensorgram of the PDMS Grating for Ultrasonic Detection

**Figure 11:**
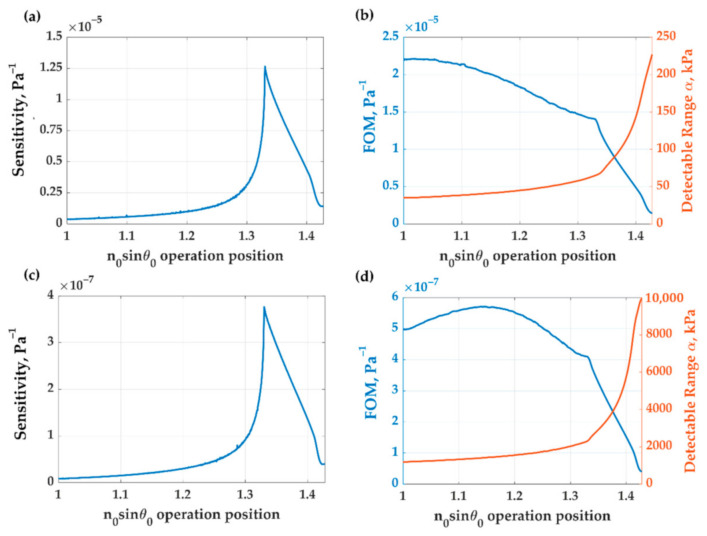
The ultrasonic detection performance in (**a**) sensitivity and (**b**) *FOM* and the detectable range, *α*, of PDMS grating in SAW mode by varying operation positions, *n*_0_*sinθ*_0_. The ultrasonic detection performance in (**c**) sensitivity and (**d**) *FOM* and detectable pressure range *α* of PDMS grating in the absence of SAW mode by varying operation positions, *n*_0_*sinθ*_0_.

Figure 11 shows the quantitative performance parameters at different operating positions of the two gratings. Grating with the SAW mode coupling has a maximum sensitivity of 1.3 × 10^−5^ Pa^−1^ at the operating position, *n*_0_*sinθ*_0_, of 1.33 as shown in Figure 11a and the maximum *FOM* of 2.2 × 10^−5^ Pa^−1^ at the operating position, *n*_0_*sinθ*_0_, of 1 as shown in Figure 11b. The highest sensitivity occurred after the critical angle, which cannot be detected by PDMS thin film. The dynamic range increased as the incident angle increased, but it was narrow as shown in Figure 11b, complying with PDMS thin film. Grating without SAW mode coupling has a maximum sensitivity of 3.8 × 10^−7^ Pa^−1^ at the operating position, *n*_0_*sinθ*_0_, of 1.33 as shown in Figure 11c and the maximum *FOM* of 5.7 × 10^−7^ Pa^−1^ at the operating position, *n*_0_*sinθ*_0_, of 1.14 as shown in Figure 11d. For product range, the both grating was look like PDMS thin film. The maximum product range approximated to 1.

### 3.3. Performance Comparison of Different Thin Film Structures

Other structures, such as surface plasmon resonance (SPR) sensor, PDMS sensor with shearing interference with the responsivity of 5.1 × 10^−7^ Pa^−1^ [18], the uniform PDMS film, as shown in Figure 3a, conventional FP structures, were also investigated to compare their performance ultrasonic detection with the proposed PDMS grating. These included a bimetallic layer [15] in which an elastic spacer was sandwiched by a 40 nm thick gold film and 7 nm thick gold film, as shown in Figure 1a. The other FP structure was an FP resonator, which an elastic spacer is enclosed by two Bragg reflectors [14] on each side comprising of alternating layers of SiO_2_ with the refractive index n_SiO2_ of 1.46 [47] and TiO_2_ with the refractive index n_TiO2_ of 2.56 [48], as shown in Figure 1b, were simulated. The spacer of both FP structures was a 25 μm PDMS film. The acoustic detection performance of each system was summarized in Table 1.

The highest *FOM* was at the normal incidence for the uniform PDMS cases and declined towards the higher incident angle, as shown in Table 1. The sensitivity was eventually zero when the operating, *n*_0_*sinθ*_0_, was beyond 1.33 since there were no FP intensity dips. The lossless uniform PDMS films and the Bragg mirrors have a similar *FOM × α*, although the sensitivity and the detection limit of the two films were different. Therefore, the *FOM × α* encapsulates the trade-off between sensitivity and the detection limit, allowing sensing platforms to be cross-compared. For lossy bimetallic layers, the resonant dip’s quality factor (Q-factor) is lower than the lossless cavity making the *FOM × α* lower than the lossless FP structure.

The PDMS grating provides FP dips after the critical angle through the diffraction process. The grating degraded the *FOM × α* performance for the incident angles before the critical angle since the light cannot form a confined FP cavity. For the illumination angles greater than the critical angle, *n*_0_*sinθ*_0_ of 1.33, the two FP gratings with/without SAW mode had similar *FOM × α* similar to the lossless FP uniform cases. In addition to the similar performance in *FOM × α*, the sensitivity of FP gratings was higher than the other structures. The sensitivity for the grating with SAW at *n*_0_*sinθ*_0_ of 1.33 was 7 times higher than the Bragg mirrors. Of course, this came with the trade-off in the detection limit. The grating can be employed as a tool for tunning the sensitivity and detection limit by changing the operating position *n*_0_*sinθ*_0_ without affecting the *FOM × α* performance. For the uniform PDMS, the sensitivity can be tuned by changing the spacer’s thickness. It not only requires a new sensor fabrication, but it can also introduce other undesirable effects, such as a change in Young’s modulus [50] of the PDMS. Another advantage of performing ultrasonic detection above the critical angle is that the lower incident angles can be fully utilized in optical imaging without sacrificing the optical imaging capability, the numerical aperture (NA). This is not the case in the uniform PDMS films.

It is interesting to point out that although the SPR mode had the lowest sensitivity level, it provided the broadest detection limit leading to the highest *FOM × α.* However, there are some drawbacks to the SPR system [51,52,53,54]. It requires a higher excitation angle [48] than the FP modes, and the metal film for the Kretschmann configuration is not transparent, obscuring the optical imaging.

## 4. Discussion on Practical Considerations

For PDMS grating fabrication, a conventional ultraviolet lithography process [55] and PDMS spin coating [56] can be applied to achieve the grating resolution and the grating period in Table 1; microscale fabrication is sufficient.

It is essential to point out that although the PDMS material is highly elastic, it has a high homogeneous thermal expansion coefficient of 300 nm/°C [57], which is corresponding to 62 kPa/°C and 2000 kPa/°C for the grating structure with SAW at *n*_0_*sinθ*_0_ of 1.33 and the grating structure without SAW at *n*_0_*sinθ*_0_ of 1.33 in Table 1. Therefore, a closed-loop temperature control unit [58] and a reference channel are suggested [35].

## 5. Conclusions

Here, we have provided a theoretical framework to explain that the transparent PDMS grating structure can give the FP modes’ diffraction mechanism allowing the embedded FP modes beyond the critical angles to be observed in optical reflectance with no need for an optical interferometer. The grating also enhances the mechanical and optical properties, providing additional compressibility for the structure when illuminated with the incident ultrasound. We have shown that some of the grating structures can couple the incident ultrasound into the surface acoustic wave mode. This can further enhance the grating compressibility. The performance parameter *FOM ×* *α* has been introduced, encapsulating the well-known trade-off between the sensor’s sensitivity and the detection limit. The *FOM × α* has enabled us to make a direct performance comparison across different platforms for ultrasonic detection. It has been demonstrated that the grating cannot change the fundamental properties of the FP mode, but it can be employed to modulate the sensitivity and the detection limit. The PDMS grating can enhance sensitivity and *FOM* by 7 times and 4 times, respectively, compared to the conventional FP structures. However, this comes with an unavoidable expense in the detection limit. It is essential to point out that the other advantages of the PDMS grating include: (1) its transparency provides a convenient integration into an optical imaging system and (2) the optical spatial resolution is not degraded by the ultrasound detection since its operating position is greater than the critical angle.

## Figures and Tables

**Table 1:** The performance of optical thin films for ultrasonic detection.

	Sensitivity (Pa^−1^)	*FOM* (Pa^−1^)	Detectable Range, *α* (kPa)	*FOM × α*
SPR sensor [49]	6.1 *×* 10^−9^	4.7 *×* 10^−8^	315,000	1.49
FP mode in PDMS thin film (*n*_0_*sinθ*_0_ = 0)	1.5 *×* 10^−^^9^	3.3 *×* 10^−7^	2720	0.90
FP mode in PDMS thin film (*n*_0_*sinθ*_0_ = 1.32)	2.1 *×* 10^−8^	5.0 *×* 10^−8^	7130	0.35
FP interferometer with bimetallic layer [15]	6.5 *×* 10^−8^	7.4 *×* 10^−^^7^	2160	0.16
FP interferometer with Bragg reflector [14]	1.9 *×* 10^−6^	3.4 *×* 10^−6^	280	0.97
Grating structure with SAW at operating point (a) (*n*_0_*sinθ*_0_ = 1.00)	3.7 *×* 10^−7^	2.2 *×* 10^−5^	35	0.77
Grating structure with SAW at operating point (a) (*n*_0_*sinθ*_0_ = 1.33)	1.3 *×* 10^−^^5^	1.4 *×* 10^−5^	67	0.93
Grating structure without SAW at operating point (b) (*n*_0_*sinθ*_0_ = 1.14)	2.0 *×* 10^−8^	5.7 *×* 10^−^^7^	1400	0.80
Grating structure without SAW at operating point (b) (*n*_0_*sinθ*_0_ = 1.33)	3.8 *×* 10^−7^	4.0 *×* 10^−7^	2388	0.95
